# Temperament, character and decision-making characteristics of patients with major depressive disorder following a suicide attempt

**DOI:** 10.1371/journal.pone.0251935

**Published:** 2021-05-20

**Authors:** Klára M. Hegedűs, Bernadett I. Gál, Andrea Szkaliczki, Bálint Andó, Zoltán Janka, Péter Z. Álmos

**Affiliations:** Department of Psychiatry, Faculty of Medicine, University of Szeged, Szeged, Hungary; Washington University, St. Louis, UNITED STATES

## Abstract

**Background:**

Multiple psychological factors of suicidal behaviour have been identified so far; however, little is known about state-dependent alterations and the interplay of the most prominent components in a suicidal crisis. Thus, the combined effect of particular personality characteristics and decision-making performance was observed within individuals who recently attempted suicide during a major depressive episode.

**Methods:**

Fifty-nine medication-free major depressed patients with a recent suicide attempt (within 72 h) and forty-five healthy control individuals were enrolled in this cross-sectional study. Temperament and character factors, impulsivity and decision-making performance were assessed. Statistical analyses aimed to explore between-group differences and the most powerful contributors to suicidal behaviour during a depressive episode.

**Results:**

Decision-making and personality differences (i.e. impulsivity, harm avoidance, self-directedness, cooperativeness and transcendence) were observed between the patient and the control group. Among these variables, decision-making, harm avoidance and self-directedness were shown to have the strongest impact on a recent suicide attempt of individuals with a diagnosis of major depressive disorder according to the results of the binary logistic regression analysis. The model was significant, adequately fitted the data and correctly classified 79.8% of the cases.

**Conclusions:**

The relevance of deficient decision-making, high harm avoidance and low self-directedness was modelled in the case of major depressed participants with a recent suicide attempt; meaning that these individuals can be described with the myopia for future consequences, a pessimistic, anxious temperament; and a character component resulting in the experience of aimlessness and helplessness. Further studies that use a within-subject design should identify and confirm additional characteristics specific to the suicidal mind.

## Introduction

Suicide represents a major public health problem worldwide as regards it takes approximately 800 000 lives per year and therefore considered as one of the leading causes of death [1]. In Europe, 10–30 attempts were reported for each completed suicides [2]. Considering that a suicide attempt is one of the most important risk factors of a completed suicide [3] and the strongest risk factor of a further suicide attempt [4], this data highlights the significance of better understanding the background of a suicide attempt.

Numerous distinct biological, social and psychological factors have been linked to suicide attempt; however, the application of multidimensional approaches could better contribute to understanding the antecedents of such a serious outcome. The present study aims to highlight some potential psychological factors characterising the status of major depressed individuals with a recent suicide attempt.

The significance of cognitive factors such as decision-making, problem-solving and autobiographical memory; personality correlates, such as impulsivity, hopelessness and particular temperament and character dimensions in suicidal behaviour have been confirmed (see [5–7]), although this list is non-exhaustive. Many studies focus on patients with a history of a lifetime suicide attempt, revealing some major trait-like vulnerability factors for suicidal behaviour (e.g. sensitivity to social stress tied to attention deficits, reward dependence; impaired problem-solving, hopelessness, impulsivity and aggression [6], decision-making [8]). However, studying the state following a suicide attempt is also important, since exploring the mind still in the period of a suicidal crisis may help us to identify individuals with an acute risk of suicide in the future.

As regards studies in which patients were treated within a maximum of two weeks following a suicide attempt, patients with major depressive disorder were characterised by higher impulsivity [9], immature defence mechanisms [10] and specific temperament and character factors (i.e. higher harm avoidance and lower self-directedness, [11]). As for cognitive aspects, research has focused on deficits in cognitive inhibition [12], pronounced cognitive impairment [13] and poor decision-making performance [14] among depressed patients with a recent suicide attempt.

These findings indicate that serious suicidal intent may emerge on the basis of pronounced neurocognitive and personality alterations. Among these variables, the study presented focused on decision-making as a cognitive function, impulsivity and Cloninger’s temperament and character factors [15] as personality components.

Decision-making is a higher-order cognitive function requiring numerous cognitive skills. Its different aspects can be measured by distinct tasks, among which decision-making in ambiguous and risky situations was observed in this research with the help of the Iowa Gambling Task’s (IGT) two versions [16, 17]. Poor overall performance and the absence of learning effect could be important indicators of suicidal behaviour associating with serotonergic impairments in the orbitofrontal cortex / ventromedial prefrontal cortex [8].

Impulsivity is a multifactorial, partly heritable construct [18], which can refer to different behavioural or personality manifestations of impaired self-regulation. Its personality aspect will be discussed in this paper. In this manner, impulsivity can refer to the lack of deliberation and persistence [19]; novelty seeking behaviour, rapid processing of information and the inability to delay gratification and to forethought before acting [20]. Although impulsivity has a complex neurobiological basis, its strong associations with the serotonergic system via 5-HT activity [18] and the dopaminergic system [21] can be highlighted.

Cloninger’s psychobiological model differentiates four temperament (harm avoidance, novelty seeking, reward dependence, persistence) and three character (self-directedness, cooperativeness, transcendence) factors [15]. Temperament is the partially heritable “disposition of a person to learn how to behave, react emotionally, and form attachments automatically by associative conditioning”, while character refers to the self-regulatory aspects of personality linking to learning and memory systems of intentionality and self-awareness and also showing heritability [22–24].

In the light of the above, the aim of the present study is to assess the possible importance of particular personality and cognitive factors of medication-free individuals with a diagnosis of major depressive disorder within 72 h following their suicide attempt. In an accompanying paper, comprehensive decision-making profile was reported in the same cohort of participants [14]. To broaden our scope, the present study also takes impulsivity, temperament and character factors into account and weighs the possible predictive power of these correlates on major depressive individuals’ status following a suicide attempt.

Concerning the results of the accompanying paper reflecting poor decision-making performance with the inability to anticipate future consequences in the patient group, importance of decision-making was hypothesized in the presented model. Relating to the observations of Eric et al. [11] examining patients with similar inclusion criteria, higher harm avoidance and lower self-directedness were hypothesized to be specific to the state of individuals who attempted suicide recently during a depressive episode. Furthermore, predictive value of these variables was assumed. Regarding impulsivity, between-group differences and its significance in the model was hypothesized.

## Methods

### Participants

Fifty-nine depressed individuals with a recent suicide attempt (mean age: 35.7, SD: 12.3; 41 female, 18 male) and forty-five healthy control subjects (mean age: 34.5, SD: 11; 25 female, 20 male) were recruited in this study.

Medication-free in-patients at the Department of Psychiatry, Faculty of Medicine, University of Szeged with a diagnosis of major depressive disorder and with a recent suicide attempt (within 72 hours) were enrolled. Individuals between 18 and 65 years of age were included. A “non-fatal self-directed potentially injurious behaviour with any intent to die as a result of the behaviour” was regarded as a suicide attempt [25]. Patients with neurological disorders, bipolar disorder, substance related disorders, schizophrenia spectrum disorders and obsessive-compulsive disorders were excluded.

Control participants were matched for age and sex, had never attempted suicide, had no psychiatric diagnosis and were free from psychiatric medication. They were recruited via convenience sampling method and their assessment took place in the outpatient exam rooms of the clinic.

The study was carried out according to the Declaration of Helsinki and was approved by the Human Investigation Review Board, University of Szeged (ethical approval number: 2443). Written informed consent was obtained from all the participants after a comprehensive description of the study.

### Measures

Diagnoses were made with the Mini-International Neuropsychiatric Interview [26].

Impulsivity was measured by the Barratt Impulsiveness Scale (BIS-10) [27]. This paper-and-pencil scale consists of 34 items and has three subscales: motor impulsivity, cognitive impulsivity and non-planning impulsivity. Temperament and character factors were assessed with the original version of the Temperament and Character Inventory [15]. This self-reporting measure consists of 240 “true” or “false” items that measure four temperament factors, harm avoidance, novelty seeking, persistence and reward dependence, and three character dimensions, self-directedness, cooperativeness and self-transcendence.

Decision-making ability was assessed with two versions of the Iowa Gambling Task (the IGT ABCD version [16] and the IGT EFGH version [17]). This computerized game captures decision-making in ambiguous and risky situations. Participants choose cards 100 times from four decks with different properties. The ABCD version contains two decks with small immediate rewards, but with tolerable future losses and two others with high immediate gains paired with significant future losses. Decks with high immediate punishments with even higher rewards and decks with small losses, but insignificant future gains are present in the EFGH version. Therefore, for a better overall outcome, acceptance of lower immediate rewards pays off with the ABCD version (it is sensitive to reward), and toleration of high immediate losses does so with the EFGH version (it is sensitive to punishment). Test performance can be evaluated based on overall net scores and sub-scores for every set of 20 choices (1–20, 21–40, 41–60, 61–80 and 81–100).

### Statistical analysis

Independent samples t-test and chi-square test were used in order to observe sociodemographic between-group differences. One-way multivariate analysis of covariance (MANCOVA) was conducted to reveal statistical differences on personality and cognitive variables between the two groups, while controlling for age as covariate regarding its possible mediating effect on the measured components [28, 29]. Bonferroni post-hoc analyses revealed adjusted between-group differences. Effect sizes were indicated by partial eta-squared. Binary logistic regression with a stepwise method of forward likelihood ratio was conducted to explore that among the observed variables, which are the strongest indicators of a suicide attempt during a major depressive episode and whether they can be included into a model with sufficient prediction value. Overall decision-making performance, impulsivity and temperament and character factors were set as covariates and age was set as indicator factors. Fitness of the model was monitored with the Hosmer-Lemeshow test.

The level of significance was set at p < 0.05. SPSS 24 [30] was used for data analysis.

## Results

Depressed individuals with a recent suicide attempt and healthy control individuals were compared with regard to age and sex. These analyses did not show significant differences (age: t(103) = 0.646, *p* = 0.519); sex: (χ^2^(1) = 1.883, *p* = 0.170).

There was a significant difference between the two groups on the combined effect of dependent variables after controlling for age (Pillai’s trace = 0.451; F(10,87) = 0.536, *p* < 0.001, ηp^2^ = 0.54). Adjusted personality and decision-making between-subject differences are presented in Table 1.

**Table 1:** Adjusted personality and decision-making differences between individuals with a diagnosis of major depressive disorder and a recent Suicide Attempt (SA) and Healthy Controls (HC).

	SA (N = 59)	HC (N = 45)	Post-hoc test			
	Mean (SD)	Mean (SD)	*F*	*df*	*p*	*ηp*^*2*^
**BIS/Total**	71.54 (12.71)	61.31 (9.78)	19.66	1	<0.001	0.17
**TCI/NS**	18.52 (6.00)	20.2 (5.71)	1.44	1	0.302	0.02
**TCI/HA**	21.91 (7.00)	12.49 (5.90)	50.58	1	<0.001	0.35
**TCI/RD**	15.41 (3.08)	16.29 (3.00)	1.65	1	0.202	0.02
**TCI/P**	4.74 (2.51)	4.71 (1.83)	0.03	1	0.955	<0.01
**TCI/SD**	23.63 (6.14)	31.13 (4.04)	47.93	1	<0.001	0.33
**TCI/C**	28.06 (6.60)	31.13 (4.04)	6.57	1	0.012	0.06
**TCI/T**	15.35 (6.97)	11.22 (5.35)	10.63	1	0.002	0.10
**IGT/ABCD**	-1.94 (21.83)	20.27 (34.32)	14.58	1	<0.001	0.13
**IGT/EFGH**	16.81 (23.67)	32.36 (42.52)	4.70	1	0.033	0.05

Abbreviations: BIS (Barratt Impulsiveness Scale), TCI (Temperament and Character Inventory), NS (novelty seeking), HA (harm avoidance), RD (reward dependence), P (persistence), SD (self-directedness), C (cooperativeness), T (transcendence), IGT (Iowa Gambling Task).

After controlling for age, stepwise forward binary logistic regression model included IGT ABCD net score, harm avoidance and self-directedness in the equation from the observed components presented in Table 1. These variables added significantly to the prediction: IGT ABCD net score (χ^2^ = 7.459; df: 1; *p* = 0.006), harm avoidance (χ^2^ = 7.502; df: 1; *p* = 0.006), and self-directedness (χ^2^ = 6.763: 0.169; df: 1; *p* = 0.009). No indication of multicollinearity was found among these variables (VIF below 2.247 for every variable in the model). The baseline model (χ^2^ = 0.816; df: 1; *p* = 0.366) had an accuracy of 54.5% overall percentage. The Hosmer–Lemeshow test (χ^2^: 9.262; df: 8; *p* = 0.321) indicates that this model adequately fitted the data. The model was significant (χ^2^: 58.108; df: 5; *p* < 0.001), explains 59.4% of the variance (Nagelkerke R^2^) and correctly classified 79.8% of cases.

## Discussion

This study observed significant decision-making, impulsivity, temperament and character differences between medication-free major depressed individuals with a recent suicide attempt and healthy control participants. Besides, it presented a model indicating that among these variables, poor decision-making on the IGT ABCD, high harm avoidance and low self-directedness were the most powerful characteristics of the patients. Furthermore, these three factors had a significant predictive value and classify 79.8% of participants correctly. Therefore, hypotheses regarding decision-making, harm avoidance and self-directedness were confirmed. Higher impulsivity was indeed present among patients; however, its assumed importance in the model was not confirmed.

The specific role of decision-making in depressed patients with a previous suicide attempt was reported earlier in several studies [31], even in comparison to major depressive individuals with no history of a suicide attempt [8]. This research also confirmed the relevance of decision-making among depressed individuals with a suicide attempt, since both IGT versions differentiated patients from control participants and IGT ABCD was included in the model presented. Importance of the reported decision-making performance was discussed in detail in the accompanying paper [14]. In summary, poor decision-making could indicate reward-sensitivity (in the IGT ABCD) or punishment-sensitivity (in the IGT EFGH). However, depressed individuals with a recent suicide attempt performed poorly on both versions and thus alternative interpretations are needed. Since decision-making is a higher-order neuropsychological function requiring numerous cognitive skills, its deficit may represent a complex cognitive disturbance. On the other hand, prediction of the near future is essential for advantageous decision-making during the IGT; thus, poor performance may be linked to the myopia for future, which is one of the major characteristics of the suicidal mind.

As regards personality components, high harm avoidance, low self-directedness, low cooperativeness, high transcendence and high impulsivity could be observed in case of depressed individuals with a recent suicide attempt.

In terms of suicidal behaviour, impulsivity could play an important role in the transition of suicidal ideations into attempt [32] and could interact with depressive state and hopelessness [33]. Impulsivity was indeed proved to be a possible characteristic of depressive state and an important indicator of suicide risk among different groups of individuals: distinct aspects of impulsivity could differentiate persons with mental disorders from healthy controls [34], depressive states from manic states [33], and differ among patients with or without a history of a suicide attempt [9, 34]. Besides, certain facets could be sensitive for suicide ideation or intent [32, 35, 36].

Since the study presented revealed higher impulsivity of major depressed individuals with a recent suicide attempt even after controlling for age, findings could be regarded as consistent with previous research. However, impulsivity was not included among the most relevant variables of the model. It is important to note that the above mentioned studies highlighted mainly between-group differences regarding impulsivity and therefore gave moderate information about its statistical power. A meta-analysis revealed small effect size of impulsivity on suicidal behaviour [37]; therefore, less robust power of this factor in the model presented also corresponds to previous findings. It could be challenging to explore the role of impulsivity on suicidal behaviour with paper-pencil tests–its behavioural indicators may represent a more relevant predictive power.

Concerning temperament and character factors, a fearful, pessimistic (high harm avoidance) temperament style and characteristics of aimlessness, blaming (low self-directedness), hostility, self-centeredness (low cooperativeness), altruism, spirituality (high transcendence) can be highlighted among depressed individuals with a recent suicide attempt. Although, interpretation of personality constellations could be more expedient, since high transcendence in itself may be indeed adaptive; however, its association with low cooperativeness and self-directedness could indicate “schizotypal” features [38].

The observation of possible interactions between high harm avoidance and low self-directedness could be also essential, since these components were included in the built model. Low harm avoidance and high self-directedness relate to resilience, the ability to maintain a healthy mental state in stressful situations [39]. The model presented shows an inverse personality constellation, indicating that adaption to different life-challenges is affected in this sample. As regards their possible importance in suicidal behaviour, high harm avoidance [5, 6, 40, 41] and low self-directedness has been reported in several studies [42, 43].

All in all, the model presented suggests that I) the inability to make decisions according to an assessment of possible future consequences, II) a pessimistic and shy temperament, and III) loss of willpower and goal-orientation may be the most powerful characteristics of major depressive individuals with a recent suicide attempt when compared to healthy controls. Furthermore, accuracy of the prediction is relatively high, meaning that patients can be differentiated with good probability from healthy persons based on these variables.

It is important to note that, although the results are mixed and non-exclusive, neural networks with serotonergic modulation seem to play an important role in these two personality factors [44, 45] and in decision-making [8], raising the possibility that changes in the serotonergic system may affect these components and link them. Besides, the orbitofrontal cortex can be highlighted as an important structure connecting both to harm avoidance [46, 47] and in decision-making functioning [8]–including functional changes during decision-making in ambiguous and risky situations [48].

These findings present the status of major depressed patients within 72 h following their suicide attempt. However, it should be discussed whether these alterations can be regarded as trait-like vulnerability factors for suicide or state-like phenomena that characterise the suicidal mind. As for poor performance on the IGT, there is no consensus on whether this is a trait- or state-dependent factor. Some studies support the former hypothesis [8]; however, analysis of a more comprehensive decision-making profile raises the possibility that more pronounced alterations may present during a suicidal crisis state [14]. As for harm avoidance, temperament factors are commonly referred to as relatively stable phenomena, but it should be highlighted that they can be modified by behavioural conditioning [22]. Besides, severity of depressive symptoms could alter harm avoidance [49–51], which means that the time of the assessment may be important even in the case of this temperament factor. Furthermore, capturing distinct states of mind could reveal differences independently of depressive symptom severity [11, 52], emphasizing the dynamic changes following a suicide attempt. If we take low self-directedness into account as well, its state-like alterations specific to suicidal behaviour can also be observed: it differentiates major depressed individuals with suicidal ideations from non-ideators [40, 53]. In conclusion, even if these factors can be regarded as trait components of suicidal behaviour, their pronounced changes may be specific to suicidal crisis states as well, showing the importance of the time of the assessment.

In summary, this study observed and discussed the relevance of distinct psychological factors among individuals with the diagnosis of major depressive disorder, who attempted suicide within 72 h. The most important finding of this study is that decision-making performance on the IGT ABCD, harm avoidance and self-directedness together could have a predictive value on attempting suicide during a depressive episode. Alterations in the serotonergic system and the orbitofrontal cortex may connect these factors. Further components related to these pathways should thus be taken into account when assessing potential risk factors for a suicidal crisis. Moreover, the fact that this model contains both cognitive and personality dimensions raises the importance of multidimensional approaches. Assessing prediction values within individuals with recent, past or no history of a suicide attempt would also be essential in order to compile clinical test-batteries sensitive for acute suicide crisis.

This study has limitations, of which the lack of a depressed inpatient control group with a past suicide attempt or without a history of a suicide attempt is the most relevant, because it limits the possibility of a suicidal state-specific interpretation. Further studies should explore other possible psychological dimension specific to suicidal crisis. In addition, recruitment of persons with a past history of a suicide attempt as control participants or a within-subject study design may also add to the existing research.
